# Extended Viral Shedding of a Low Pathogenic Avian Influenza Virus by Striped Skunks (*Mephitis mephitis*)

**DOI:** 10.1371/journal.pone.0070639

**Published:** 2014-01-29

**Authors:** J. Jeffrey Root, Susan A. Shriner, Kevin T. Bentler, Thomas Gidlewski, Nicole L. Mooers, Jeremy W. Ellis, Terry R. Spraker, Kaci K. VanDalen, Heather J. Sullivan, Alan B. Franklin

**Affiliations:** 1 United States Department of Agriculture, Wildlife Services, National Wildlife Research Center, Fort Collins, Colorado, United States of America; 2 United States Department of Agriculture, Wildlife Services, National Wildlife Disease Program, Fort Collins, Colorado, United States of America; 3 Department of Microbiology, Immunology, and Pathology, Colorado State University, Fort Collins, Colorado, United States of America; Texas A&M Veterinary Medical DIagnostic Laboratory, United States of America

## Abstract

**Background:**

Striped skunks (*Mephitis mephitis*) are susceptible to infection with some influenza A viruses. However, the viral shedding capability of this peri-domestic mammal and its potential role in influenza A virus ecology are largely undetermined.

**Methodology/Principal Findings:**

Striped skunks were experimentally infected with a low pathogenic (LP) H4N6 avian influenza virus (AIV) and monitored for 20 days post infection (DPI). All of the skunks exposed to H4N6 AIV shed large quantities of viral RNA, as detected by real-time RT-PCR and confirmed for live virus with virus isolation, from nasal washes and oral swabs (maximum ≤10^6.02^ PCR EID_50_ equivalent/mL and ≤10^5.19^ PCR EID_50_ equivalent/mL, respectively). Some evidence of potential fecal shedding was also noted. Following necropsy on 20 DPI, viral RNA was detected in the nasal turbinates of one individual. All treatment animals yielded evidence of a serological response by 20 DPI.

**Conclusions/Significance:**

These results indicate that striped skunks have the potential to shed large quantities of viral RNA through the oral and nasal routes following exposure to a LP AIV. Considering the peri-domestic nature of these animals, along with the duration of shedding observed in this species, their presence on poultry and waterfowl operations could influence influenza A virus epidemiology. For example, this species could introduce a virus to a naive poultry flock or act as a trafficking mechanism of AIV to and from an infected poultry flock to naive flocks or wild bird populations.

## Introduction

Wild waterfowl and shorebirds (orders Anseriformes and Charadriiformes) are generally considered the primary natural hosts of avian influenza viruses (AIVs) [1]. For obvious reasons, much of the attention associated with AIVs has been focused on these and other avian taxa, while the potential role of wild mammals in the epidemiology of these viruses has received limited attention [2]–[4].

The presence of mammalian wildlife on poultry farms has been suggested to be a risk factor associated with the dissemination of LP AIV among poultry operations in the eastern U.S. [5]. In addition, wild animals such as skunks (Family Mephitidae) and opossums (presumably *Didelphis virginiana*), were suggested as a possible source of pandemic H1N1 2009 virus to three mammalian species in a zoological garden in San Diego [6]. Therefore, it is imperative that studies are conducted to address the potential concern of mammal involvement with influenza A virus epidemiology, especially those mammals commonly associated with anthropogenically modified habitats.

Of particular interest, some species in the mammalian family Mustelidae and the closely related family Mephitidae appear to be susceptible to a number of influenza A viruses, including AIVs (Table 1). For example, an H4N10 AIV was detected in mink farms (presumably *Mustela vison*; aka *Neovison vison*) in Sweden [7], an H1N2 influenza A virus was detected in mink in a captive facility in the mid-western U.S. [8], the HP H5N1 Asian strain AIV was detected in a stone marten (*Martes fonia*) in Germany [9], and the pandemic 2009 H1N1 virus has been detected in domestic ferrets (*Mustela putorius furo*) [10], an American badger (*Taxidea taxus*), a black-footed ferret (*Mustela nigripes*) [6], and striped skunks (*Mephitis mephitis*) [11]. Additional mammalian associations with influenza A virus have been reviewed within the past few years [3], [4]. As these viruses are further scrutinized in mammals, more associations are likely to be discovered in the future, and key mammalian families in AIV ecology may be identified.

**Table 1:** Reported natural influenza A virus exposures detected in the mammalian families Mustelidae and Mephitidae.

Common name	Scientific name	Virus	Morbidity/Mortality	Location	Reference
American mink	*Mustela vison* (*Neovison vison*)	H4N10	Approximately 3000 deaths	Mink Farms/southern Sweden	[7]
		HP H5N1	Euthanized due to aggressive disease	Presumably feral/southern Sweden	[15], [31]
		H1N2	Death of 10 animals per day for weeks	Mink Ranch/Midwestern U.S.	[8]
Stone marten	*Martes fonia*	HP H5N1	Euthanized due to severe morbidity	Wild animal/Germany	[9]
Domestic ferret	*Mustela putorius furo*	2009 H1N1	One euthanized due to lack of response to treatment; some treated	Ferret Shelter/Kentucky	[10]
			All recovered	House pets/Oregon	[10]
			Respiratory infection/fate unknown	House pet/Oregon	[10]
			One died, three presumably recovered	House pets/Nebraska	[10]
American badger	*Taxidea taxus*	2009 H1N1	Euthanized due to severe morbidity	Zoo animal/California	[6]
Black-footed ferret	*Mustela nigripes*	2009 H1N1	Mild clinical illness	Zoo animal/California	[6]
Striped skunk^a^	*Mephitis mephitis*	2009 H1N1	Eight died (two known to have an influenza infection); complications from additional pathogens	Wild skunks near a mink farm/British Columbia	[11]

a Note: Until recently, skunks were considered to be member of the family Mustelidae. However, recent genetic work has placed them in a new family designated as Mephitidae [32].

Striped skunks are known to be peri-domestic in certain situations, often living at the human-animal interface and associated with farms. For example, striped skunk maternal dens and resting sites are commonly located under farmstead buildings [12] and this species is often considered a pest on farms when they inhabit farm buildings [13]. Of interest, striped skunks are known to consume considerable amounts of animal-matter during the winter and spring, such as small mammals and the eggs and young of ground-birds [13]. This feeding behavior, especially if one considers fecal-laden waterfowl eggs, could represent a mechanism of AIV transmission to striped skunks. Further, striped skunks have a large geographic distribution, ranging throughout most of the continental U.S. into various regions of Canada and northern Mexico [14].

Most mammals have not been thoroughly scrutinized for their role(s), if any, in the epidemiology of LP AIVs. However, the peri-domestic nature of select mammalian species could make them a potential risk for trafficking these viruses. In addition, certain families of mammals, such as Mustelidae and allies, in which several wild and domestic species appear susceptible to multiple subtypes of influenza A virus [6]–[8], [10], [11], [15], warrant more detailed studies on their potential roles in virus trafficking and transmission. For these reasons, the objective of this study was to assess viral shedding potential and the course of infection of a common peri-domestic mammalian species, the striped skunk, experimentally infected with a LP H4N6 AIV that is commonly found in wild waterfowl in the U.S. [16].

## Methods

### Ethics Statement

Animal experiments were approved by the Institutional Animal Care and Use Committee of the National Wildlife Research Center (NWRC), Fort Collins, CO, USA (Approval number 1797). The study animals were captured on private and public lands with permission from the landowners and stewards with the appropriate state collection permit.

### Study animals

Eight striped skunks were live captured in Tomahawk live traps (81.3×25.4×30.5 cm; Tomahawk Live Traps, LLC, Hazelhurst, WI, USA) in Larimer County, Colorado, USA. The skunks were chemically anesthetized (intramuscular injection of a 5∶1 ratio of ketamine/xylazine) in the field and transported to the National Wildlife Research Center (NWRC) in custom modified transport devices designed for gas anesthesia. Upon arrival at the NWRC, the animals were re-anesthetized with isoflurane for processing (e.g., blood collection and nasal samples, microchip application, parasite dusting, etc.). Serum and nasal swab samples were stored at −80°C until analyses were conducted. The animals were maintained in 2.4×2.4×3.0 m outdoor animal pens during a quarantine period (≥10 days). Following quarantine, all skunks were moved into ABSL-2 facilities and housed in 7′×7′×8′ pens. The animals were randomly assigned as treatment (n = 7) or control (n = 1) animals. All animals were supplied with a den box, a water bowl, a food bowl, a litter box, and an enrichment toy. Food (omnivore diet; Mazuri, Purina Mills, LLC, St. Louis, MO) and water were replenished each day. The skunks were comprised of an equal sex ratio (4 male and 4 female) and had an average mass of 2.25 kg (range 1.01–4.78), suggesting these animals were <1 year to several years old.

### Experimental Infection

On day 0 of this experiment, all animals were chemically anesthetized using the ketamine/xylazine methods outlined above, and nasally inoculated with approximately 10^6^ EID_50_ of a LP AIV (A/Mallard/CO/P70F1-03/08(H4N6)) (passage history detailed elsewhere [17]) delivered in 1 mL of BA-1 viral transport media (see [18] for formula). The control skunk received a 1 mL mock inoculation of BA-1.

For daily sampling from 1–14 days post infection (DPI), striped skunks were lightly chemically immobilized with slight modifications of the doses described previously. Daily processing was the same for each animal and consisted of nasal washes that induced sneezes (1 mL of BA-1), oral swabs, and fecal swabs (i.e., feces collected from pens or litter boxes). All swabs were stored in 1 mL of BA-1 diluent. Samples were stored on ice packs and were subsequently transferred to −80°C freezers immediately following the conclusion of daily processing. A single skunk was again processed on 16 DPI because it was still shedding relatively large quantities of viral RNA on 14 DPI. On 20 DPI, all skunks were anesthetized (see methods above) and euthanized with an intravenous injection of Beuthanasia-D Special (Schering-Plough Animal Health Corp., Union, NJ) following collection of nasal washes, oral swabs, and blood samples. Necropsies were performed to collect select tissues for real-time reverse-transcription polymerase chain reaction (RRT-PCR) and pathological analyses.

### Necropsy and Tissue Processing

The following tissues were typically fixed in 10% buffered formalin, preserved in ethanol, embedded in paraffin, sectioned at 5 µm, and stained with hematoxylin and eosin for histological examination: heart, spleen, liver, kidney, lung, brain, small intestine, bladder, large intestine, trachea, stomach, and adrenal gland. In addition, nasal turbinates, trachea, lung (upper and lower lobes), and colon were collected into vials with 1 mL BA-1. Samples were homogenized for extractions as previously described for testing by RRT-PCR [18]. All animal carcasses were incinerated following necropsies.

### Laboratory Testing

Nasal washes, oral swabs, and fecal swabs were tested in duplicate by RRT-PCR for viral RNA detection and quantification. RNA was extracted using the MagMAX-96 AI/ND Viral RNA Isolation Kit (Ambion, Austin, TX). Primer and probe sequences specific for the influenza type A matrix gene [19] were used with one modification to the probe; the fluorescent quencher, TAMRA, was replaced with a non-fluorescent quencher, BHQ-1. RRT-PCR was performed in duplicate following slight modifications of a previously developed protocol [20]. Each RRT-PCR reaction contained 5 µL 5× buffer, 1.0 µL enzyme mix, and 0.8 µL of dNTP mix included in the Qiagen® One-Step RT-PCR kit (QIAGEN Inc., Valencia, CA) along with 3.75 mM MgCl_2_, 0.266 units/µL RNase Inhibitor (Promega Corp., Madison, WI), 10 pmol of each forward and reverse primer, and 0.12 µM probe in a total volume of 17 µL. Eight microliters of extracted RNA template was added to bring the final reaction volume to 25 µL. RRT-PCR was performed in an ABI 7900HT thermocycler (Life Technologies Corp., Carlsbad, CA) with the following conditions: 50°C for 30 min, 95°C for 15 min, and 45 cycles of 94°C for 1 sec and 60°C for 30 sec. Calibrated controls with known viral titers were also extracted and analyzed with RRT-PCR to construct standard curves for downstream analyses. Viral RNA quantities from samples were extrapolated from the four-point standard curves and are presented as PCR EID_50_ equivalents/mL. Positive samples were defined as those yielding a two-well positive amplification with a Ct value of ≤38 and suspect positive samples were defined as those yielding a two-well positive amplification with a Ct value of >38. Virus isolation was conducted on nasal wash and oral swab samples (1–10 DPI only) in SPF embryonated chicken eggs following published protocols [21].

Pre-exposure and 20 DPI serum samples were analyzed with standard AGID tests [22], [23] and by ELISA with the FlockCheck® Avian Influenza MultiS-Screen Antibody Test Kit (IDEXX Laboratories, Inc, Westbrook, ME). For ELISA purposes, we did not use a stringent sample-to-negative (S/N) cutoff ratio, which is a ratio of the sample absorbance to the mean negative control absorbance. Rather, we utilized the observed differences in S/N ratios between pre-exposure and 20 DPI sera to assess serologic activity in striped skunks. Because neither assay has been thoroughly evaluated on striped skunk sera, we present results from the two independent assays for purposes of comparison.

## Results

### Nasal Shedding

**Figure 1:**
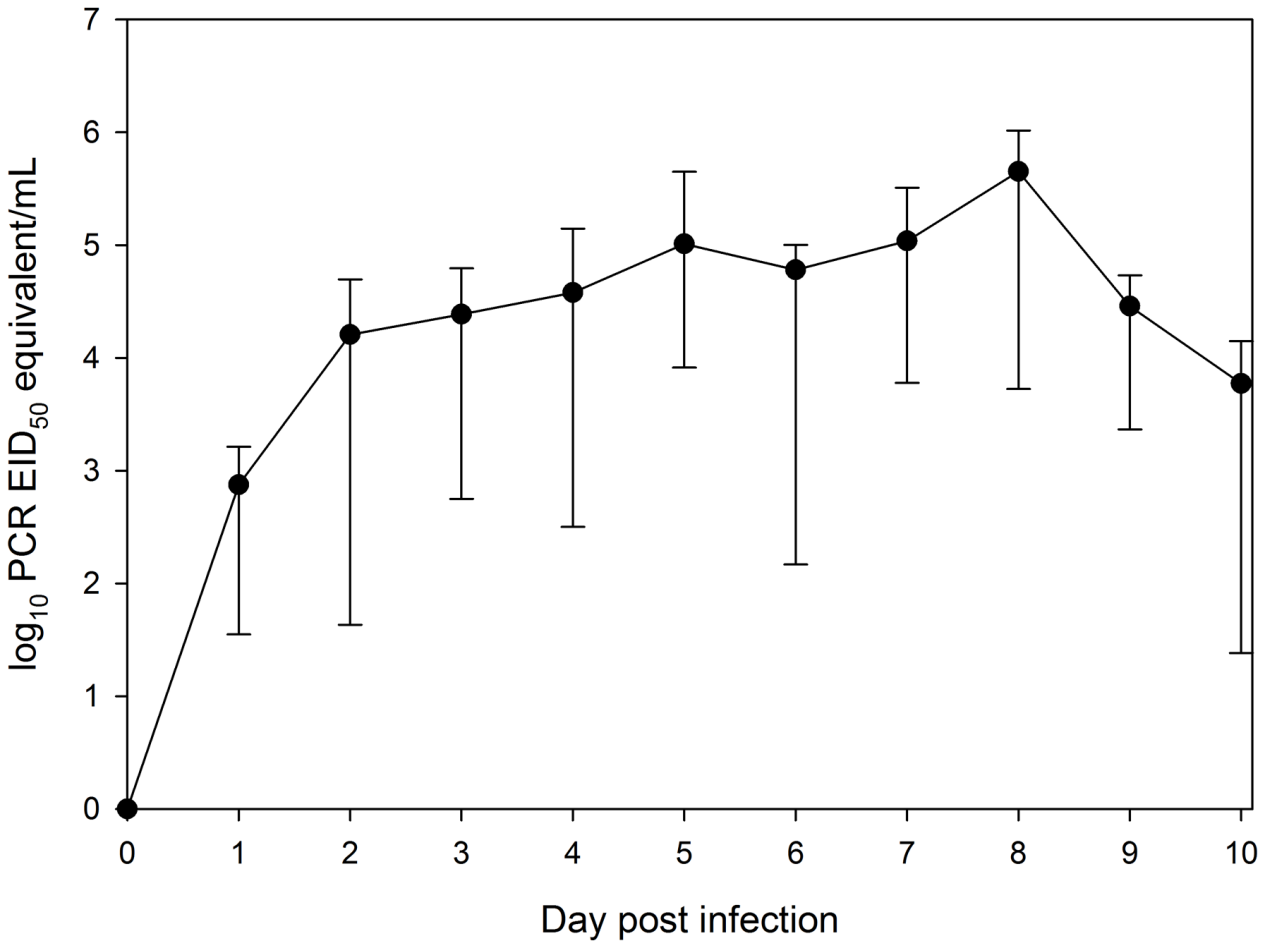
Mean nasal shedding of avian influenza virus RNA of striped skunks (*Mephitis mephitis*) experimentally infected with a low-pathogenic avian influenza virus. Shedding was assessed from nasal washes by RRT-PCR. Results are presented as log_10_ PCR EID_50_ equivalents/mL. Vertical bars represent the maximum and minimum quantities detected on a given day.

All skunks typically yielded productive sneezes, presumably excreting upper respiratory fluids with BA-1. Generally nasal washes yielded at least 500 µl of the initial BA-1 dispensed into the nasal cavities of skunks, although this varied by individual. Subsequently, all inoculated animals showed suspect or greater evidence of nasal shedding of AIV RNA by 1 DPI (Figure 1). Nasal shedding peaked on 8 DPI for 6 of 7 skunks, yielding an average of 10^5.65^ PCR EID_50_ equivalent/mL (range = 10^3.72^ to 10^6.02^; Table 2). By 14 DPI, four skunks were negative for viral RNA, two skunks yielded suspect positive results, and a single skunk yielded a positive result of 10^3.03^ PCR EID_50_ equivalent/mL. The latter individual was positive on 16 DPI and suspect positive on 20 DPI. In addition, one other individual that was suspect positive on 14 DPI remained suspect positive on 20 DPI (this individual was not sampled on 16 DPI). The nasal washes from all other individuals were negative by 20 DPI (Table 2). Aside from one exception, all nasal wash samples testing positive by RRT-PCR were also confirmed positive for live virus by virus isolation during 1–10 DPI.

**Table 2:** Nasal shedding of striped skunks (*Mephitis mephitis*) experimentally infected with an avian influenza virus (H4N6).

	DPI																
Animal	0	1	2	3	4	5	6	7	8	9	10	11	12	13	14	16	20
A	--a	2.90*^b^	4.70*	4.61*	5.14*	5.65*	4.85*	5.51*	5.70*	4.73*	4.02*	3.54	4.24	4.45	3.03	1.85	S^c^
B	--	3.15*	4.15*	4.58*	4.51*	4.89*	4.94*	5.19*	6.02*	4.48*	3.67*	3.35	2.44	3.36	S	nt	S
C	--	1.73	3.82*	3.39*	3.69*	4.33*	5.00*	4.65*	5.42*	4.72*	4.15*	3.99	2.38	1.96	S	nt	--
D	--	2.92*	4.11*	4.79*	4.83*	4.98*	4.97*	4.97*	5.42*	3.56*	S*	S	S	2.13	--	nt	--
E	--	S	S	2.75*	2.50*	3.91*	2.17^nt^	3.78*	3.72*	3.37*	3.12*	S	2.10	--	--	nt	--
F	--	3.21*	4.44*	4.20*	4.07*	4.56*	4.13*	4.99*	5.12*	4.35*	3.51*	S	S	S	--	nt	--
G	--	2.69*	3.24*	4.04*	3.85*	4.47*	4.72*	4.64*	5.97*	4.55*	3.88*	3.65	S	S	--	nt	--
Control^d^	--	--	--	--	--	--	--	--	--	--	--	--	--	--	--	nt	

a Nasal shedding was assessed via nasal washes by RRT-PCR. Results are presented as log_10_ PCR EID_50_ equivalents/mL. A dash “--” indicates that no viral RNA was detected. The term “nt” indicates the sample was not taken or there was insufficient sample volume to conduct the test.

b * = Live virus confirmed by virus isolation in eggs. Only samples from 1–10 DPI were tested.

c Suspect positive: two wells positive but Ct>38.

d Individual was a mock inoculated control skunk housed in the same animal room, but in a separate pen.

### Oral Shedding

**Figure 2:**
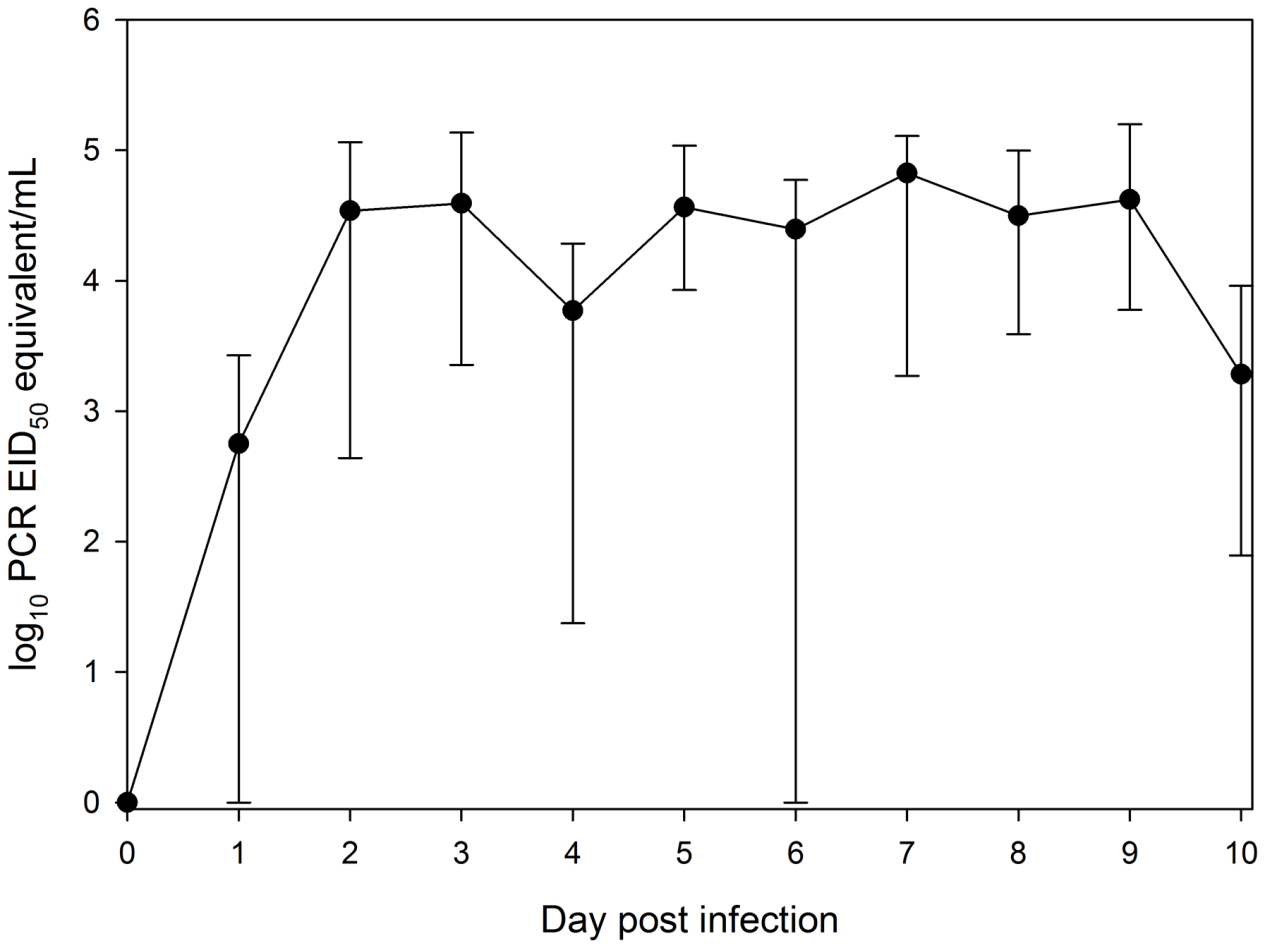
Mean oral shedding of avian influenza virus RNA of striped skunks (*Mephitis mephitis*) experimentally infected with a low-pathogenic avian influenza virus. Shedding was assessed from oral swabs by RRT-PCR. Results are presented as log_10_ PCR EID_50_ equivalents/mL. Vertical bars represent the maximum and minimum quantities detected on a given day.

Over one-half of skunks yielded evidence of oral shedding of AIV by 1 DPI, and all seven experimentally infected skunks showed evidence by 2 DPI (Figure 2). Oral shedding peaked, in general, on 7 DPI with an average of 10^4.82^ PCR EID_50_ equivalent/mL (range = 10^3.26^ to 10^5.10^; Table 3). However, shedding quantities were flat across a number of days such that the peak quantity on 7 DPI was similar to those obtained on 5–9 DPI. The highest quantity swab occurred on 9 DPI and was 10^5.19^ PCR EID_50_ equivalent/mL. By 11 DPI, two individuals were negative for viral RNA, one individual yielded suspect positive results, and four individuals yielded positive results (Table 3). At 14 DPI, only one individual yielded a positive oral swab. At 20 DPI, all oral swabs yielded negative results. Aside from few exceptions, all oral swab samples testing positive by RRT-PCR during 1–10 DPI were also confirmed positive for live virus by virus isolation.

**Table 3:** Oral shedding of striped skunks (*Mephitis mephitis*) experimentally infected with an avian influenza virus (H4N6).

	DPI															
Animal	1	2	3	4	5	6	7	8	9	10	11	12	13	14	16	20
A	2.89^a^	4.29*^b^	4.92*	1.98*	4.43*	3.16*	3.26*	3.99*	4.01*	3.96*	3.37	--	--	3.12	2.75^c^	--
B	1.82*	4.44*	4.22*	4.19*	4.62*	3.94*	5.00*	4.62*	3.77*	3.08*	2.87	3.48	--	--	nt	--
C	2.61	4.56*	3.51*	1.37*	5.03*	4.32*	4.33*	4.50*	4.78*	2.74	3.23	--	2.98	--	nt	--
D	3.42*	5.06*	4.29*	3.28*	4.67*	4.69*	4.98*	4.43*	4.39*	2.94	S^d^	--	S	--	nt	--
E	S	2.64*	3.35*	3.03*	3.92*	--	3.69*	3.78*	3.77*	3.00*	--	--	--	--	nt	--
F	S	4.53*	5.13*	4.28*	4.02*	4.51*	5.10*	3.58*	5.19*	1.89*	--	--	--	--	nt	--
G	--	3.80*	4.01*	3.48*	4.02*	4.77*	5.04*	4.99*	4.42*	2.70*	2.89	2.67	S	--	nt	--
Control^e^	--	--	--	--	--	--	--	--	--	--	--	--	--	--	nt	--

a Oral shedding was assessed via oral swabs by RRT-PCR. Results are presented as log_10_ PCR EID_50_ equivalents/mL. A dash “--” indicates that no viral RNA was detected. The term “nt” indicates the sample was not taken or there was insufficient sample volume to conduct the test.

b * = Live virus confirmed by virus isolation in eggs. Only samples from 1–10 DPI were tested.

c A single animal was sampled on 16 DPI (nt = sample not taken).

d S = Suspect positive: two wells positive but Ct>38.

e Individual was a mock inoculated control skunk housed in the same animal room, but in a separate pen.

### Fecal Shedding

Suspect viral RNA was detected in the feces of striped skunks beginning at 2 DPI. With two exceptions on 3 and 8 DPI, all RNA detections were at the suspect level. The two exceptions were low level positives of ≤10^2.04^ PCR EID_50_ equivalent/mL. No individuals yielded evidence of viral RNA in feces after 10 DPI. Considering the fecal samples were collected from pens and not directly from the animals, these results should be interpreted with caution (see Discussion).

### Viral RNA Detection in Tissues

Select tissues (nasal turbinates, trachea, upper lung, lower lung, and colon) were collected during necropsy on 20 DPI for RRT-PCR analyses. Nasal turbinates yielded positive results in one individual and suspect positive results in a second individual. All other tissues were negative.

### Serology

All inoculated striped skunks yielded evidence of a serological response in their convalescent sera at 20 DPI, with an average difference between S/N ratios from ELISA runs of −0.54 (range = −0.82 to −0.23). For comparison, the difference in the S/N ratios of the control skunk was 0.11. AGID results were consistent with those obtained from ELISAs, as all inoculated skunks were scored as either positive (n = 4) or strong positive (n = 3). The control skunk yielded no evidence of a serological response.

### Pathology

Significant histological lesions were not observed between the control and infected skunks exposed to avian influenza virus. Lesions found in control and infected skunks were limited to nutritional (hepatic lipidosis) conditions and incidental parasitic (nematodes and cestodes) infections.

## Discussion

With few exceptions [11], [24], striped skunks have received very limited attention for their roles in the ecology of influenza A viruses. However, the viral shedding observed in this study coupled with their often synanthropic habits [25], large potential for mobility [26], and their documented destruction of waterfowl nests [27], suggests that this species possesses a combination of characteristics that make it strong candidate for avian influenza virus dissemination.

Raccoons (*Procyon lotor*) are also often considered peri-domestic and have previously been studied for susceptibility and possible transmission of AIV [2], [17]. Unlike raccoons, striped skunks were documented to shed large quantities of viral RNA via the nasal route. Part of this discrepancy could be associated with methodological differences. For example, nasal swabs were used to assess nasal shedding in other studies [2], [17], while this study exclusively used nasal washes during experimental procedures. Given that a nasal wash typically elicits one or more sneezes, this procedure likely has the potential to detect viral RNA from deeper within the respiratory tract. Therefore, this sampling method may be more indicative of the potential shedding of the virus from the host through nasal mucous or a sneeze. Additional differences among these two species may be related to dose, inoculation route, and the subtypes used, as one study intranasally inoculated raccoons with 10^5.0^ EID_50_ of an H4N8 virus [2], and a second relied on animals to naturally consume an H4N6 virus through water and food [17]. Overall, the effect of inoculation dose on subsequent shedding in striped skunks is unclear at this time, but may alter the duration of the infectious period and shedding patterns. In addition, natural infections may be influenced by repeated exposures to large quantities of virus.

Although nasal shedding was the most prominent route of shedding in striped skunks, relatively high levels of oral shedding were also noted. Oral shedding peaked, on average, on 7 DPI, one day earlier than peak average nasal shedding (Figure 2). The highest oral swab detected yielded 10^5.19^ PCR EID_50_ equivalent/mL. This is in contrast to what we have detected in raccoons, with positive results inconsistently detected on various DPI at low quantities [17]. Thus, skunks yielded a higher, more prolonged, and more consistent oral shedding than raccoons. In addition, peak oral shedding was noted much earlier in raccoons as compared to striped skunks. Tracheal shedding of an H3N8 virus has been observed in a small number of striped skunks during a previous study; however, the levels of shedding were not quantified [24]. During the present study, oral swabs were always taken prior to nasal washes. As such, the viral RNA detected in the oral cavity is likely representative of that which could be found naturally and not due to a recently induced sneeze or cough.

We also detected low levels of viral RNA in feces above the suspect level on rare occasions. This occurred in 1 animal during 3 DPI (10^1.65^ PCR EID_50_ equivalent/mL) and on a second during 8 DPI (10^2.04^ PCR EID_50_ equivalent/mL). All remaining “positive” fecal results were at the suspect level (n = 27; 2–10 DPI). However, these data should be interpreted with caution. For example, positive fecal samples could be a result of a sneeze or oral secretion contaminating the fecal material and may not represent actual fecal shedding. However, considering that other mammalian species have been shown to replicate AIV in their intestinal tracts [28], along with the rapid cessation of suspect fecal shedding observed in this study (no evidence of any fecal shedding after 10 DPI) while both nasal and oral shedding were still occurring (Tables 2 and 3), these data suggest that striped skunks may shed very low quantities through feces. This is consistent with previous work, as rectal shedding of an H3N8 virus (titers were not reported) has been observed previously in striped skunks [24].

Noticeable signs of disease were not observed in this study. In contrast, striped skunks naturally infected with pandemic H1N1 virus were thought to have died due to complications with severe pneumonia [11]. However, those skunks were also co-infected with Aleutian disease virus and bacterial infections, both of which could have contributed to disease complications and subsequent death [11]. An American badger infected with pandemic H1N1 virus experienced aggressive, irreversible disease, but only mild clinical illness was noted in a black-footed ferret [6]. No disease symptoms were reported for striped skunks experimentally infected with H1N1 and H3N8 subtypes [24]. Thus, it is apparent that the disease caused by influenza A viruses in these taxa varies widely by viral strain and the mammalian species that it infects.

It has been previously suggested that the presence of mammalian wildlife on poultry farms may be a risk factor associated with the movement of LP AIV among commercial operations in the eastern U.S. [5]. The current study suggests that striped skunks might add to this risk, as striped skunks appear to shed much larger quantities of AIV as compared to raccoons (*Procyon lotor*) [2], [17]. Previously, researchers have suggested that infected raccoons could transport an influenza A virus from a rural area to agricultural operations [2]. A similar scenario may be plausible for striped skunks. As such, skunks could contaminate poultry or waterfowl feed or water with respiratory or oral secretions while visiting a farm. Several attributes of AIV could facilitate transmission of this virus to striped skunks from contaminated water at poultry farms or areas where wild birds congregate: 1) AIVs can remain viable in water or moist organic materials for long periods of time [29], 2) water is a known source of AIV transmission to at least one wild mammalian species [17], and 3) AIV has been isolated from the drinking water of an experimentally infected striped skunk [24]. Considering that skunks shed large to moderate quantities of viral RNA for up to or beyond two weeks post infection, mammal-to-mammal transmission of AIV has been documented via close contact [7], and bird-to-mammal transmission has been experimentally documented [30], the aforementioned scenario might be possible. Additional studies are needed to assess the ecological transmission mechanisms of AIVs in striped skunks and to assess natural exposures of these viruses in skunks and allies.
